# Dynamic switching between intrinsic and extrinsic mode networks as demands change from passive to active processing

**DOI:** 10.1038/s41598-020-78579-6

**Published:** 2020-12-08

**Authors:** Frank Riemer, Renate Grüner, Justyna Beresniewicz, Katarzyna Kazimierczak, Lars Ersland, Kenneth Hugdahl

**Affiliations:** 1 Mohn Medical Imaging and Visualization Centre, University of Bergen and, Haukeland University Hospital, Bergen, Norway; 2 Department of Radiology, Haukeland University Hospital, Bergen, Norway; 3 Department of Physics and Technology, University of Bergen, Bergen, Norway; 4 Department of Biological and Medical Psychology, University of Bergen, Bergen, Norway; 5 Department of Clinical Engineering, Haukeland University Hospital, Bergen, Norway; 6 Division of Psychiatry, Haukeland University Hospital, Bergen, Norway

**Keywords:** Neuroscience, Cognitive neuroscience, Neuro-vascular interactions, Visual system

## Abstract

In this study we report on the relationship between default and extrinsic mode networks across alternating brief periods of rest and active task processing. Three different visual tasks were used in a classic fMRI ON–OFF block design where task (ON) blocks alternated with equal periods of rest (OFF) blocks: mental rotation, working memory and mental arithmetic. We showed the existence of a generalized task-positive network, labelled the extrinsic mode network (EMN) that is anti-correlated with the default mode network (DMN) as processing demands shifted from rest to active processing. We then identified two key regions of interest (ROIs) in the supplementary motor area (SMA) and precuneus/posterior cingulate cortex (PCC) regions as hubs for the extrinsic and intrinsic networks, and extracted the time-course from these ROIs. The results showed a close to perfect anti-correlation for the SMA and Precuneus/PCC time-courses for ON- and OFF-blocks. We suggest the existence of two large-scale networks, an extrinsic mode network and an intrinsic mode network, which are up- and down-regulated as environmental demands change from active to passive processing.

## Introduction

Ever since the discovery and identification of the default mode network (DMN) by Marcus Raichle and colleagues^1–3^, the study of network interactions has been a major issue in imaging neuroscience^4^. This has in particular concerned intrinsic interactions and anti-correlations between the DMN and other resting-state networks, and task-positive networks which are activated during active task-processing^5–8^. Other studies have focused on the decomposition of the DMN into sub-networks typically activated in task-processing situations^9–13^. At around the same time as the discovery of the DMN, Duncan and Owen^14^ suggested that regions in the dorsolateral and anterior cingulate frontal cortex were activated during multiple processing demands. Duncan and Owen^14^ described their discovery as a "regional specialization of function within prefrontal cortex" (p. 475), which generalised across a variety of cognitive tasks and situations. The initial discovery by Duncan and Owen^14^ was later replicated and extended by Fedorenko et al.^15^, who found a common network structure across seven memory, executive, and attention tasks, and by Duncan^16^ who now labelled these activations a multiple demand (MD) system. Adding to this, Hugdahl et al.^17^ performed a retrospective analysis using nine separate fMRI experiments and found a generalized task non-specific network, which was activated across all nine tasks. This network involved the SMA/anterior cingulate, lateral prefrontal cortex, and inferior parietal lobule. Hugdahl et al.^17^ labelled this network the extrinsic mode network (EMN) to distinguish it from the intrinsic DMN. It is therefore clear that not only is there a linked set of network activations during resting periods, which collectively could be called non-specific task-negative networks, but also a set of similar non-specific task-positive networks. This set of networks has been labelled by different groups as the multiple demand system^16^, frontal-lobe network^14^, or extrinsic mode network^17^. We will use the term "extrinsic mode network (EMN)" in the following to describe this generalised task-positive network. Although the EMN and other task non-specific networks show overlapping features with task-specific networks, there are also essential differences, in particular that the EMN and other general-domain networks^15,16^ are observed in a wide range of tasks across cognitive domains^17^ -while task-specific networks are more specific to a particular cognitive domain, such as attention or executive functions^10,12^. The interaction between task non-specific and task specific networks is however complex and not fully understood. There are overlapping activations in fronto-parietal areas between the network categories, which could be perceived as nodes in task-specific networks that are also existing in task non-specific networks, and may modulate the strength of the latter depending on the nature of the task. An important question is how the DMN and EMN networks interact with regard to dominant up- and down-regulations across time when environmental demands repeatedly change from active to passive task-processing, i.e., change from engagement to rest and vice versa, on a short-term basis. Most studies of the relationship between task-negative and task-positive networks are either conducted during prolonged resting-periods, or by having subjects solve a single task. A single-task paradigm is usually performed by only addressing a single cognitive domain^1,10,18^, like working memory or attention as examples. An experimental set-up like this would however not capture the question of the dynamics of resting-state and non-specific task-positive network interactions, where tasks change from one processing period to another across the experimental session. Hugdahl et al.^19^ suggested an experimental proxy to the everyday switching between periods of task engagement alternated with periods of rest, where tasks moreover will differ in terms of cognitive domain and processing load from one processing period to another. In the confines of an fMRI experiment, this can be obtained by alternating task-presence and task-absence periods, using a traditional fMRI block-design^20^. In such an experiment, ON- and OFF-blocks represent active versus passive processing periods, respectively. Such an approach was taken by Hugdahl et al.^19^ who used an auditory dichotic listening task^21–23^ with ON-blocks and pseudo-random presentations of three different cognitive tasks involving perception, attention, and executive function that were interspersed with OFF-blocks and no task present. The results showed statistically significant anti-correlations between the DMN and EMN, particularly in the inferior frontal and posterior cingulate cortex regions. Moreover, EMN up-regulations at the transition from an OFF- to an ON-block were steeper and more prolonged, than the corresponding up-regulation of the DMN in the transition from an ON- to an OFF-block. The study by Hugdahl et al.^19^ was however confined to the auditory modality, and had the different cognitive domains embedded within a single task, the so called "forced-attention dichotic listening task"^22^. Thus, it is not known if a similar pattern of interactions between the DMN and EMN would hold for; (a) the visual modality, (b) when splitting the cognitive domains across tasks, and (c) expanding the tasks to more complex cognition, like number arithmetic, working memory, and mental rotation. The three tasks chosen represent three cognitive domains typically encountered during an ordinary day. To activate visuo-spatial processing, a mental rotation task^24^ was chosen because this task is shown to provide significant fronto-parietal activations^25–27^. A working memory task was chosen because working memory is a central cognitive concept, which includes attention and executive control in addition to short-term memory^28,29^, and would thus be a valid proxy for the varying processing demands during an ordinary working-day. A mental arithmetic task with adding numbers was chosen because it draws on a common cognitive ability encountered every day, the ability for mental arithmetic and to manipulate numbers. Previous research has shown that this task in isolation produces reliable activation in inferior frontal cortex and anterior cingulate^30,31^. In addition to whole brain analysis, we chose two region-of-interests (ROIs) for comparisons and correlations between the DMN and EMN networks, respectively. For the DMN we chose the precuneus as an ROI, since this region is consistently activated during DMN up-regulations^5,32^. For the EMN we chose the intersection of anterior cingulate (ACC) and supplementary motor area (SMA), since this region is consistently activated during periods of EMN up-regulation^15,17^. The justification for the choice of the SMA as the key hub for the ROI analysis was also to allow a direct comparison to our previous study with auditory tasks^19^. A second justification was to contrast this region with the precuneus region for the DMN. The aim of the present study was therefore to provide an extension of the Hugdahl et al.^19^ study by including different tasks, and cognitive processing strategies, while staying within the same ON–OFF experimental approach as the one used in Hugdahl et al. ^19^. Considering the current replication crisis in neuroscience and psychology^33^, an extended replication is a necessary first step for establishing a solid factual basis for new findings.

## Results

### Behavioural data

Mean response accuracy was calculated as the ratio of correct responses to overall number of responses and expressed as a hits-ratio. For the mental rotation task, the hits ratio was 0.560, for the working memory task it was 0.769 and for the mental arithmetic task it was 0.843. The corresponding Cohen's d values were: 0.55, 0.91, and 1.84 for the mental rotation, working memory, and mental arithmetic tasks, respectively. Following a standard interpretation of corresponding effect sizes, all three tasks showed medium to strong effect size. Mean response latency, also known as reaction-time (RT), for the three tasks were 404.2 ms (SD 143.0) for the mental rotation task, 168.4 ms (SD 56.5) for the working memory task and 218.3 ms (SD 38.0) for the mental arithmetic task. These results confirm that the subjects had understood and performed the tasks as expected.

### fMRI data

Mean framewise displacement (FD) indices for all subjects were = 0.19 ± 0.08 mm. 17 out of the 47 subjects had FDs ≥ 0.20 and were excluded for a re-analysis to compare to the results of the whole data-set. The activation maps for the re-analysis of the subset confirmed the results of the whole-dataset. We therefore decided not to remove any subjects due to motion. Adding the estimated motion parameters as a regressor did also not affect any of the results significantly.

**Figure 1:**
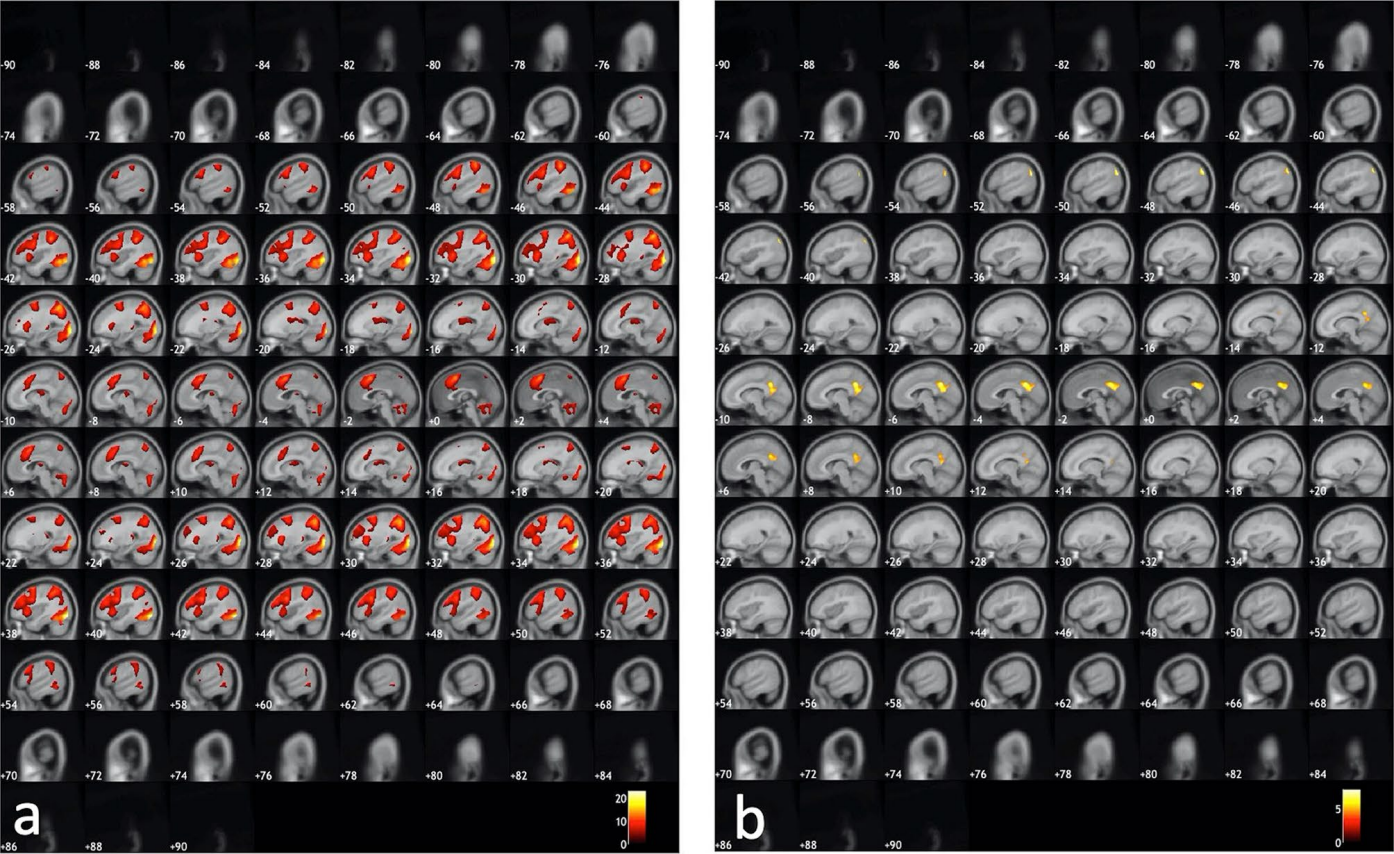
Activations that passed the .05 FWE-corrected significance threshold, shown on sagittal slices of the MNI-template. The panel (**a**) to the left shows activations obtained during ON-blocks contrasted with activations obtained during OFF-blocks (ON–OFF). The panel (**b**) to the right shows activations obtained with the contrast flipped, i.e. obtained during OFF-blocks contrasted with activations obtained during ON-blocks (OFF–ON). See “Results” for further details.

Figure 1 shows the results from the inclusive conjunction analysis of mean joint activations across the three tasks, *p* < 0.05, family-wise-error (FWE) corrected, with significant activations for the ON–OFF contrast seen in the left-hand panel and corresponding activations for the OFF–ON contrast seen in the right-hand panel. A cluster size of minimum 20 voxels was used and slices are shown with 2 mm spacing.

**Figure 2:**
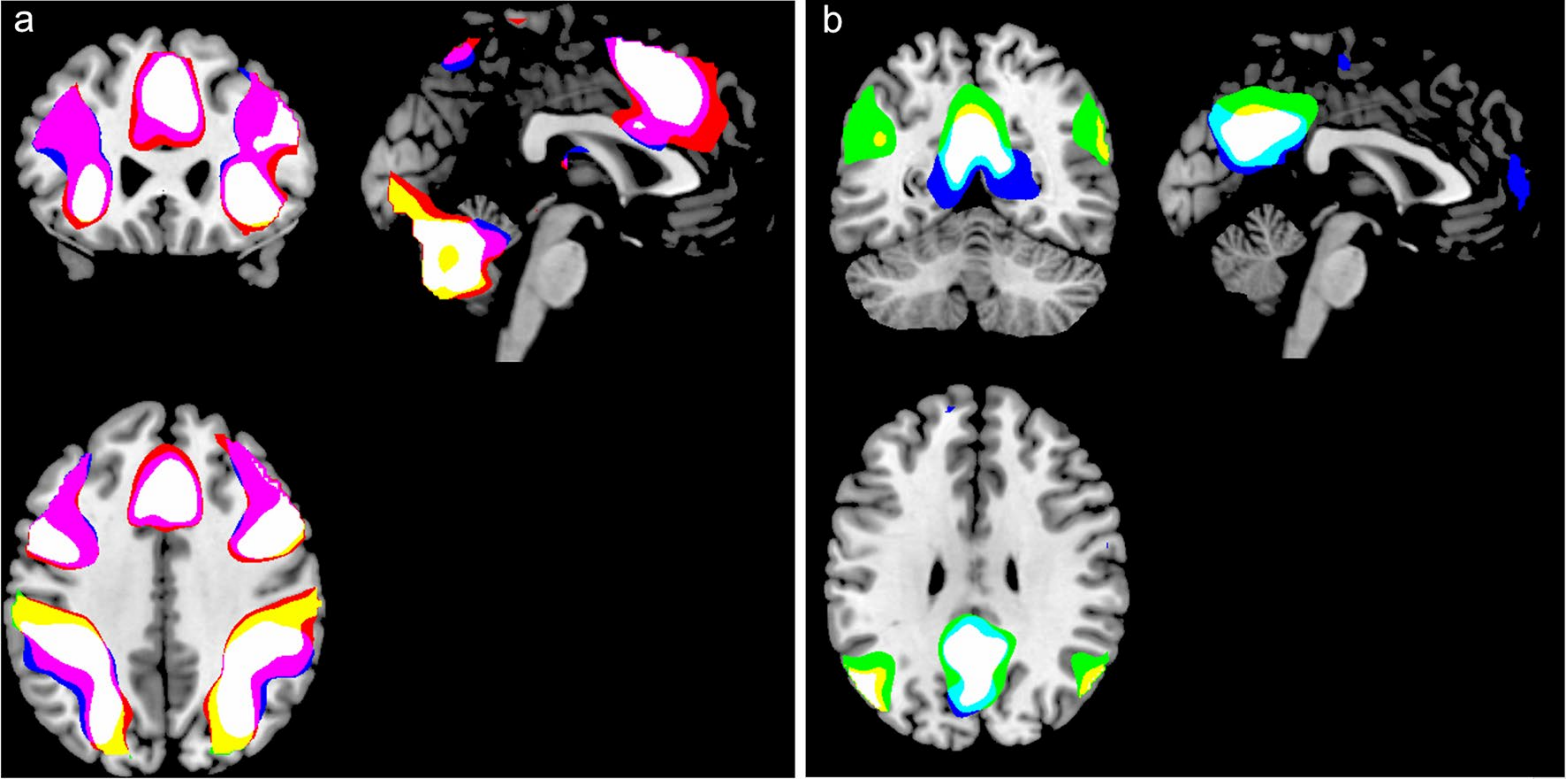
Activations for the three separate tasks overlaid on top of each other demonstrating significant overlap. The panel (**a**) to the left shows activations obtained during ON-blocks contrasted with activations obtained during OFF-blocks (ON–OFF). The panel (**b**) to the right shows activations obtained with the contrast flipped, i.e. obtained during OFF-blocks contrasted with activations obtained during ON-blocks (OFF–ON). For both panels (**a**) and (**b**), activation caused by the working memory task is shown in red, activation caused by the mental rotation task is shown in blue and activations caused by the mental arithmetic task is shown in green. Common activations across all three tasks are correspondingly indicated in white colour. Common activations for the mental rotation and working memory tasks are indicated in violet colour. Common activations for the mental rotation and mental arithmetic tasks are indicated in cyan colour, and common activations for the working memory and mental arithmetic tasks are indicated in yellow colour.

The ON–OFF contrast produced significant, *p* = 0.05, FWE-corrected activations in the right SMA (MNI coordinates: × 4, y 14, z 50), right inferior occipital gyrus (× 32, y − 85, z − 32), right angular gyrus (× 30, y − 64, z 44), left superior parietal lobule (x − 26, y − 58, z 54), right and left precentral gyrus (× 46, y 10, z 32, and x − 46, y 4, z 30, respectively), left anterior insula (x − 30, y 18, z 6) and left middle frontal gyrus (x − 26, y − 4, z 50). All t-values were > 7.46, critical 0.05 t-value was = 4.40 with 447 df. The OFF–ON contrast produced corresponding activations in the left precuneus/posterior cingulate gyrus (PCC) (x − 6, y − 54, z 32/ × 0, y 52, z 31), left and right angular gyrus (x − 48, y − 70, z 36 and × 50, y − 68, z 30) and in the right central operculum (× 38, y − 14, z 18). All t-values were > 4.97 with a critical 0.06 t-value = 4.40. Figure 2 shows the results split for the three tasks, with activations overlaid on the MRICron anatomical template (ch2.better.niftii) as different layers and colours: As seen in Fig. 2, the main findings from the overall conjunction analysis were confirmed in the separate analyses, with essentially similar patterns of activations for all three tasks. Activation caused by the working memory task is shown in red in Fig. 2, activation caused by the mental rotation task is shown in blue and activations caused by the mental arithmetic task is shown in green. Common activations across all three tasks are correspondingly indicated in white colour. Common activations for the mental rotation and working memory tasks are indicated in violet colour. Common activations for the mental rotation and mental arithmetic tasks are indicated in cyan colour, and common activations for the working memory and mental arithmetic tasks are indicated in yellow colour.

Analysing the contrasts separately for each task also yielded activations beyond what was seen in the conjunction analysis across tasks. For the ON–OFF contrast, the mental rotation task yielded additional activation in the right thalamus (× 24, y − 30, z 4), and the mental arithmetic yielded additional activations in right and left thalamus (× 26, y − 30, z 2, and x − 24, y -30, z 0, respectively). For the OFF–ON contrast, the corresponding additional activations for the working memory task were seen in the left and right lingual gyrus (x − 24, y − 44, z − 8 and × 30, y − 38, z − 12, respectively), in the left medial and lateral superior frontal gyrus (x − 2, y 58, z 8 and x − 16, y 38, z 52, respectively) and in the right posterior insula (× 38, y − 12, z 16). For the mental arithmetic task, the corresponding additional activations were seen in the right cerebellum (× 30, y − 74, z − 40), left middle temporal gyrus (x − 62, y 34, z 48) and in the left superior frontal gyrus (x − 22, y 34, z 48).

**Figure 3:**
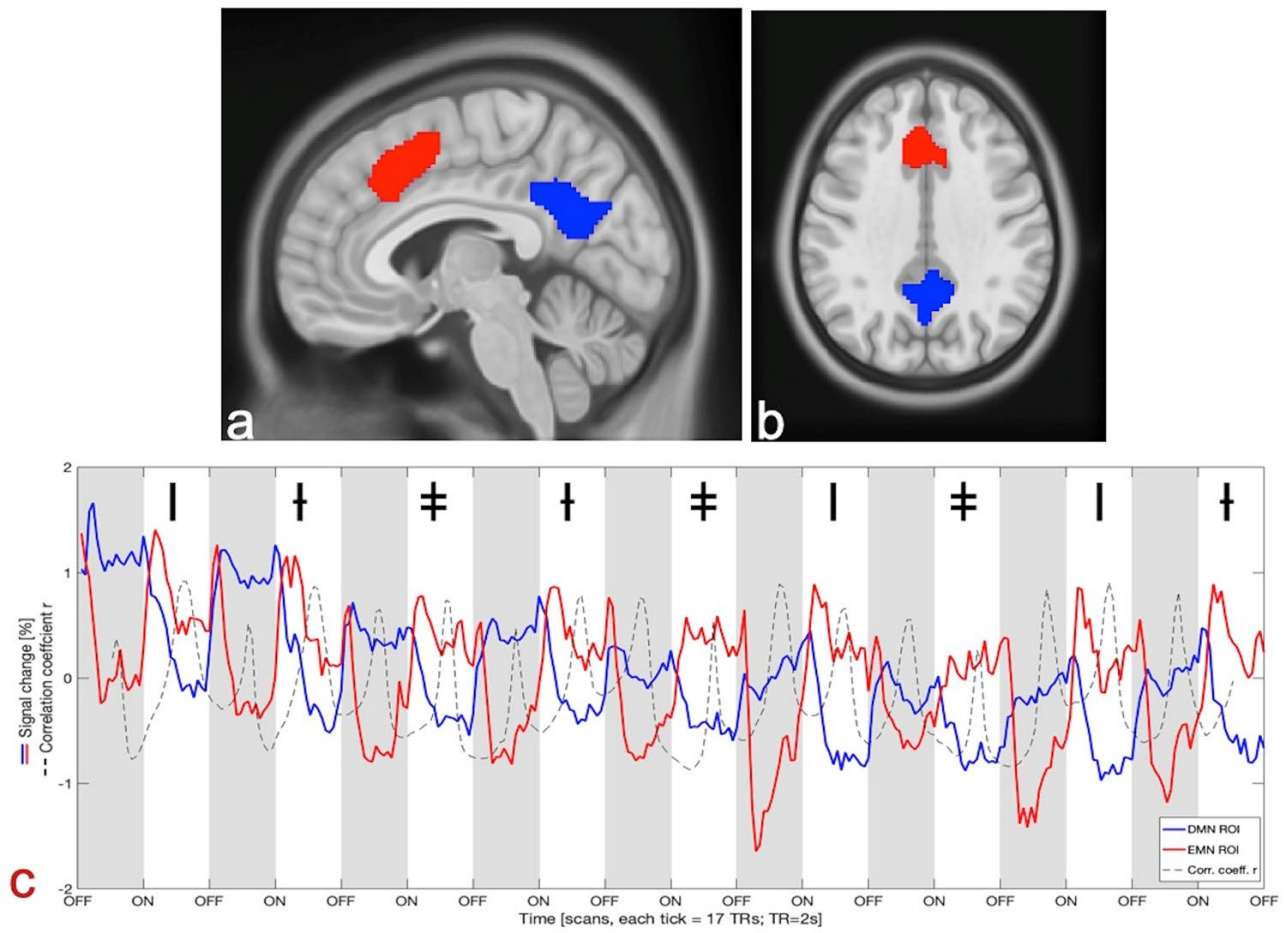
Sagittal (**a**) and axial (**b**) views of the ROIs shown on the MNI-T1 template. The DMN ROI is shown in blue and the EMN ROI in red. The ROIs were used for the time-series extraction shown in (**c**): The tasks alternated randomly between mental rotation (marked with the symbol I), working memory (marked with the symbol Ɨ) and mental arithmetic (marked with the symbol ǂ). Signal change is shown as percentage of total signal change and keeping in line with the colour coding for the ROIs as in panels (**a**) and (**b**). Onsets and offsets of the task and rest periods are shown on the x-axis as ON and OFF respectively with OFF-blocks also being marked in grey. The black intermittent line in panel (**c**) shows the Pearson correlation coefficients continuously calculated on the mean of a 17 s sliding-window through the whole session.

We then identified overlapping activations for all three tasks in the SMA and precuneus/PCC and defined them as new ROIs for the subsequent analysis, as these regions are key nodes in the EMN and DMN networks, respectively^2,17^. The ROIs are shown on top of the anatomy template in the upper panel of Fig. 3a,b. The mean time-course from these regions was extracted separately for ON- and OFF-blocks across the whole scanning session (lower panel of Fig. 3c). Windowed correlation coefficients were calculated using a time-window of 17 s with 50% overlap (half the length of one ON- or OFF-block). Development of correlation coefficients across time is shown as an intermittent black line in Fig. 3c. The black line in Fig. 3c demonstrates that there was a strong negative correlation at the beginning and end of each task-related ON-block (mean Pearson r-coefficients at points of inflection was − 0.56 ± 0.21) and corresponding strong positive correlation was seen at approximately the middle of each task ON- and rest OFF-periods (mean peak Pearson R's was + 0.68 ± 0.19).

## Discussion

We demonstrated that switching between resting and active processing periods results in two distinct networks that were up- and down-regulated as environmental demands changed from passive to active. In this respect, the present results resemble the results from a similar study where the processing tasks were auditory in nature^19^. We therefore conclude that the existence of a generalised task-positive network is not dependent on sensory modality. The most conspicuous similarities between the current and the Hugdahl et al.^19^ study were in the overlapping activations in the SMA and insula regions. There were however also differences between the previous Hugdahl et al.^19^⁠ and the current study. In the Hugdahl et al.^19^⁠ study, activations during ON-blocks were seen in auditory regions in the temporal lobes, while the present study showed activations in visual regions in the occipital cortex, as expected due to difference in task modality. Other differences were in the pre-central motor area, because a motor task was used in the current study, and in the superior parietal and middle frontal gyrus. These latter differences most likely refer to the wider range of cognitive tasks used in the current study in addition to targeting the visual modality. For the DMN, the most conspicuous similarities between the studies were found for the cingulate cortex and the precuneus, while differences were found for the superior and middle frontal gyri, and the angular operculum. Again, these differences probably reflect variation in DMN spatial extension as environmental demands vary, since OFF-task activation is affected by ON-task activations^34^. The behavioural results confirmed that the subjects were performing the tasks required throughout the scanning session. A second finding was that the up-regulation of the task-positive network was independent of the specifics of the task, and as seen in Figs. 2 and 3, i.e. generalised across the tasks. In this respect, the present results are in line with the findings of Fedorenko et al.^15^ and Duncan^16^ pointing to the existence of a task non-specific network, which we have labelled the EMN, following the nomenclature from Hugdahl et al.^17^. There are several notable similarities between the current findings, the previously suggested EMN network and the findings reported in the Fedorenko et al. and Duncan papers: Most notably are the agreement of activations in the SMA, superior parietal lobule, inferior and middle frontal gyrus, and the precentral gyrus. A major difference between our and the previous studies is in the tasks being used to elicit these activations, and the interpretation of the significance of the findings as extending beyond an attention model as a mediating factor. Considering that the DMN in essence is a task-negative network^2,5,35,36^ being up-regulated in periods of absence of specific processing demands, it is an intrinsic mode network. We now provide more evidence that the brain may alternate between an intrinsic and extrinsic mode of function, corresponding to the dominating environmental demand, with the intrinsic mode network dominating during task-absence, and the extrinsic mode network dominating during task-presence as has been previously suggested^18,19,35^. Interestingly, our results also show that the two networks are sometimes positively correlated, suggesting that the relationship between DMN and EMN may be more complex than we originally thought^19^. We would also like to suggest that intrinsic and extrinsic mode networks operate at a superordinate level with regard to domain-specific networks, like the salience network^10,11,37^, dorsal attention network^3,38^, or central executive network^12,39^.

We focused on the precuneus/PCC and ventral SMA as ROIs when extracting the time-courses representing the extrinsic and intrinsic mode networks, respectively, since the SMA was overlapping in activation for all three cognitive tasks, and has previously been implicated in a variety of cognitive operations, including visuo-spatial processing, working memory, and mental arithmetic^40^. The precuneus region was chosen because it was strongly activated during OFF-blocks, and has previously been shown to be implicated in the default mode network^1,11,41,42^. Thus, the time-course dynamics seen in Fig. 3 from the SMA and precuneus ROIs is taken as a proxy for the up- and down-regulation of the corresponding extrinsic and intrinsic mode networks. We suggest that the role of the SMA, and in particular the ventral portion, is overlapping with the pre-SMA. While Fig. 3 shows a clear inverse up- and down-regulation between the two ROIs for the alternating task and rest periods, the signals are not anti-correlated at all time (see dotted line in Fig. 3). During the middle of the task-blocks, a positive correlation can be seen, despite the rate of change of the two signals seeming static (a plateau or equilibrium is reached in the middle of the task block). This is in conflict with findings by Fox et al.^5^, who used a paradigm of three alternating rest periods (visual fixation, eyes open and eyes closed) instead of alternating rest and active periods, as employed in our study. The results of the Fox et al. study are therefore not directly comparable with the current results since the paradigm used in their study did not involve periods of active task-processing. Moreover, as seen in Fig. 3, the positive correlations seem to be driven by the EMN BOLD-response (red line) beginning to dip slightly after an initial up-regulation at the transition points. This could imply a corresponding fading of concentration focus across active blocks, or a habituation effect due to task repetitions. Further investigation is necessary to sort out these inconsistencies. In addition, to capture a good surrogate for the EMN and DMN networks, we used relatively large ROIs, which could have introduced a partial volume effect with a different network that does contribute to the positive correlation observed at times. Our mean framewise displacement^43,44^ indices are both smaller than the “lenient” (FD > 0.5 mm), as well as the “stringent” (FD > 0.2 mm) thresholds discussed by Power et al.^44^. Similarly, the popular resting-state functional connectivity MRI (rs-fcMRI) toolbox CONN only gives an outlier warning if FD > 0.5 (https://web.conn-toolbox.org/fmri-methods/preprocessing-pipeline)^45^. Therefore, corrections such as temporal masking by censoring or interpolation of the data are not likely to give any significantly different results in our study. In addition, spurious activations are less likely to impact the results in a task-based paradigm as opposed to a rs-fcMRI study. Nevertheless, we performed a simple test for influence of motion by removing 17 of the total of 47 subjects that had FDs ≥ 0.2. The results yielded no major differences to the activations patterns observed from the full dataset. More complex censoring and interpolation approaches may be more thorough but were not deemed necessary given the low mean framewise displacement.

The conjunction analysis revealed in addition the other following areas activated during ON-blocks: The angular gyrus, superior parietal lobule, anterior insula, and the middle frontal gyrus. All these regions are implicated in higher-order cognition, including mental arithmetic, visuo-spatial processing, mental rotation and working memory^27,46–48^. In addition, the occipital activation, which most likely reflects the fact that the tasks were all visual in nature. Likewise, the activation bilaterally in the pre-central region would be related to the motor-response and button-pressing. The OFF-blocks were characterised by activations in the angular gyrus and central operculum, which are regions previously implicated in the DMN^32,49^. Looking at Fig. 2, which shows areas with overlapping and non-overlapping activations for the three tasks reveals a remarkable similarity in activation patterns across all three tasks during ON-blocks (see white-coloured areas in Fig. 2). This is evident for all activations, except for the left inferior parietal lobule, which was more strongly activated to the mental rotation and working memory tasks than in the mental arithmetic task. A similar pattern emerged from looking at the overlapping activations for OFF-blocks, i.e. whether activations during resting periods associated with the three tasks differed (see white-coloured areas in Fig. 2). Again, as for ON-block periods, the pattern of activations for the OFF-blocks were quite similar across resting periods, especially for the precuneus with centre of gravity showing similar overlapping activations. Activation related to the mental rotation task did however extend in the inferior axis, and was also seen with a small area in the medial-ventral frontal lobe. The patterns of overlapping activations differed however for ON- and OFF-blocks when it comes to overlapping areas for only two tasks. For ON-blocks, the mental rotation and working memory tasks showed overlapping activations (shown in violet colour in Fig. 2) to a greater extent than the other task-combinations, which was not the case for OFF-blocks. For OFF-blocks, most of the activations were overlapping for all three tasks, with mental arithmetic (shown in green colour) in addition activating the middle temporal and superior frontal gyri. These areas are implicated in working memory tasks^50^ and may be overshooting from the ON-periods for this particular task. The ON–OFF contrast resulted in unique activations in the thalamus for mental rotation and mental arithmetic tasks, as well as in left lateral superior frontal gyrus. Thalamus activations have previously been reported for both mental arithmetic and mental rotation^51,52^ and may be related to the role of basal ganglia in number processing and visuo-spatial processing in general. Figures 1 and 2 clearly demonstrate that joint activations across tasks, both during ON- and OFF-blocks were not perfect, showing unique single-task activations, as well as joint activations for combinations of two tasks. This suggests that the EMN and other non-specific general-domain networks may be dynamically modulated depending on environmental demands, such that task-specific nodes in the network may transiently be modulated by dynamically stretching or shrinking. We suggest a similar dynamic modulation of the DMN, seen during OFF-blocks, in that its shape and level of up-regulated modulation depends both on previous and anticipated environmental challenges, which has been shown in several studies (see Bruckner et. al^4^ for a recent review of DMN activations). The absence of unique activations for the mental arithmetic task during ON-blocks, not subsumed either under the conjunction of all three tasks (white color in Fig. 2a), or overlapping with activations associated with the working memory task (yellow color in Fig. 2a), needs further investigation since it is in contrast to the additional activations during OFF-blocks associated with the mental arithmetic task (green color in Fig. 2b). We suggest that the reason for the absence of unique mental arithmetic activations during ON-blocks is that this task contains a transient working memory component in addition to a pure arithmetic component, making it a doubly-demanding task. The additional extended activations during resting periods (OFF-blocks) associated with the mental arithmetic task would in contrast imply that an arithmetic task results in stronger up-regulation of the DMN (at least defined as an extended area) than the other two tasks, which in turn could indicate that this task is more resource demanding, despite being subsumed during ON-blocks. We now suggest that a way of defining task-demands could be defined by its extent from "side to side", i.e. how much of rebound a task causes during intermittent rest-periods.

In conclusion, the present results have shown the existence of a generalized task-non-specific network, which is upregulated during periods of active task-processing, but is essentially independent of the specifics of the task. By following the nomenclature introduced by Hugdahl et al.^17^ we have labelled this network the Extrinsic Mode Network (EMN), as an extrinsic mode network in contrast to the default mode network, which is an intrinsic mode network, and should perhaps also be labelled so. Secondly, building on our previous research with auditory tasks^19^, the current results have shown that similar network dynamics exist for visual tasks, which in isolation has been shown previously^1,18^. We now show that this also occurs across different tasks in a single scanning session. For these reasons, we now suggest that a standard, classic, fMRI block-design with alternating task- and rest-periods, may be an experimental proxy for alternating rest-periods combined with task processing requirement periods that we encounter in the course of an ordinary day in life. Having said that, it should be noted that such periods in reality will vary with regard to intensity, frequency and length as environmental demands vary, and will also vary between individuals.

## Methods

### Subjects

The subjects were 47 healthy, adult individuals, 27 males and 20 females, mean age 34.9 years (SD 13.9). The subjects were recruited in the Bergen city area through general, open announcements. Absence of any psychiatric and neurological disorder was an inclusion criteria and information was obtained from self-reports. Information about education and mathematical skills level was not formally collected. There is however no reason to assume major differences in education among the subjects, since they all were young adults having been through the obligatory 10 year school system in Norway. Visual acuity and colour-vision was not formally tested, but the behavioural results show that these factors could not have been a confounding factor.

### Stimuli and design

**Figure 4:**
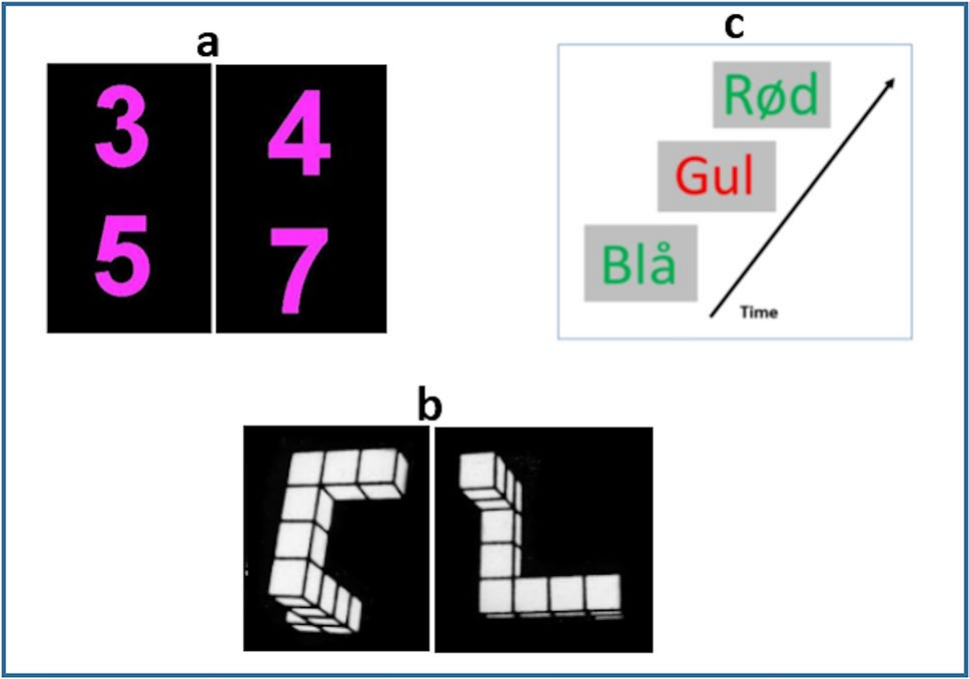
Examples of the stimuli used in the three cognitive tasks. (**a**) Shows examples of the digit pairs shown on each trial: The left part of the example shows two digits that do not match the target since the sum of the two is < 11. To the right are shown two numbers that match the target since the sum is 11, and the subject should then correspondingly press the response button. (**b**) Shows two of the 3D objects that should be compared for similarity: The example shows two object shapes that are the same but rotated horizontally with regard to each other. The subject should press the response button in this example if the shape is the same. (**c**) Shows an example of the 2-back Stroop working memory task in Norwegian, where the instruction is to press the button when the colour of the word matches with the colour shown two items before.

There were three different cognitive tasks and stimuli: a mental arithmetic task, a working memory task and a mental rotation task. The tasks were each repeated three times in 30 s ON-blocks in a random order, alternated with 30 s OFF-blocks (Fig. 4).

The stimuli were constructed with graphics software (MS-paint and Corel PaintShop) and stored as bitmap-files for presentation through high-resolution LCD HD goggles (NordicNeuroLab Inc., Norway, https://nordicneurolab.com/) mounted to the MR head-coil. The timing, duration and sequencing of the stimuli, as well control of overall timing parameters of the experiment was done in the E-Prime (Psychology Software Tools, Inc., USA, https://pstnet.com/) software platform. Synchronization timing of presentation of the stimuli with acquisition of the MR data was done through a NordicNeurolab SyncBox (NordicNeuroLab Inc., Norway, https://nordicneurolab.com/). There were nine ON-blocks with task presentations (three repetitions of each task), alternated with nine black screen OFF-blocks without task-presentations. The OFF-blocks had a fixation-cross in the middle of the visual field, and the subjects were instructed to keep their eyes open during OFF-blocks. Each ON- and OFF-block lasted for 60 s, making up a total of 18 min.

The mental arithmetic task consisted of two digits that could vary between the digits 1–9, written on top of each other, in purple font against black background (see Fig. 4a for examples). Each pair of digits was presented for 1200 ms with a blank gap of 300 ms between trials and 20 pseudo-random trials in each ON-block. The instruction to the subject was to mentally add the two numbers and press a hand-held button whenever the sum was "11". There were five target trials randomly interspersed among the 20 trials (25%), with the restriction of not allowing two or more consecutive targets by random selection. Thus, there were 15 target trials in total.

The mental rotation task (Fig. 4b) was tailored on the classic Shephard and Metzler mental rotation task^24^ with images of two 3D non-configurative objects in white against black background, that were displayed on each trial. The task of the subject was to decide by pressing the hand-held button whether the two shapes were two different objects, or two shapes of the same object, which were rotated horizontally with regard to each other (rotation varied between 20° and 180°). Each presentation trial lasted for 1500 ms, with 20 trial presentations during an ON-block, and where the two shapes were the same on 10 trial presentations and different on 10 trial presentations. The instruction was to press on "same", thus there were a total of 30 targets across the Mental rotation task.

The working memory task (Fig. 4c) was an n-back task with presentations of incongruent Stroop colour-words, like the word "red" written in blue ink. The instruction to the subject was to remember the colour of the word presented two items back (2-back paradigm), and to press the hand-held when the item currently seen in the goggles matched the one presented two items back. This task adds an executive function process to the working memory process, making it cognitively more demanding. It has previously been shown to discriminate between healthy and depressed patients^28^ and reliably elicits demands for working memory processing. Each presentation lasted for 1500 ms, with 20 presentations in total, and with five (25%) pseudo-randomly interspersed target trials where the two displays matched. Thus, there were a total of 15 targets for the working memory task.

### Behavioural data statistics

Behavioural data for the three tasks were available for 46 participants, with one data set missing. Behavioural data were automatically collected and stored by the E-prime software, for later statistical analysis of response accuracy and response latency (RT ms).

For all three tasks, response accuracy and RT was evaluated statistically for means, standard deviations, and hits ratio of correct responses to false alarms. Hits ratios were converted to Cohen's d-statistic for effect sizes in each task.

### Procedure

The subjects were first interviewed for body implants such as pace-makers and any signs of claustrophobia after they were presented with the informed consent form to sign. Females were in addition asked about pregnancy, which was an exclusion criterion. The study was approved by the Regional Committee for Medical Research Ethics in Western Norway (2014/1641/REK Vest). The study was conducted according to the Declaration of Helsinki regarding ethical standards and in agreement with the good clinical practice framework in accordance with all rules and guidelines at our institution for human research. Subjects who agreed to take part in the study were then informed about the experiment and shown examples of the stimuli and familiarized with the tasks to be presented when in the scanner, before being placed in the scanner. Written informed consent was obtained from all participants. To facilitate direct communication with the MR-technicians in the control-room, a balloon was placed on the chest, which should be squeezed in case of an emergency. There was also direct audio contact between the scanner chamber and the control room. The E-Prime stimulus program was run from a stand-alone PC and operated by a research assistant.

### MR scanning and data acquisition

The experiment was conducted on a 3 T Magnetom Prisma MR scanner (Siemens Healthcare, Germany). An anatomical T1-weighted image was acquired prior to the functional imaging with the following sequence parameters: MPRAGE 3D T1-weighted sagittal volume, TE/TR/TI = 2.28 ms/1.8 s/900 ms, acquisition matrix = 256 × 256 × 192, field of view (FOV) = 256 × 256 mm^2^, 200 Hz/px readout bandwidth, flip angle = 8 degrees and total acquisition duration of 7.40 min. 2D gradient echo planar imaging (EPI) was performed with the following parameters: TE/TR = 30 ms/2 s, 306 volumes in time, acquisition matrix = 64 × 64, slice thickness = 3.6 mm, 35 slices, FOV = 230 × 230 mm^2^.

### fMRI data processing

All pre-processing and analysis were performed in Matlab 9.5 (the MathWorks, Natick, MA) using SPM12-r7219 (the Wellcome Centre for Human Neuroimaging, UCL, London, UK, https://www.fil.ion.ucl.ac.uk/spm/). Pre-processing consisted of rigid-realignment, normalisation to MNI space and smoothing with an 8 × 8 × 8 mm^3^ Gaussian kernel. After pre-processing, first level analysis of all subjects was performed using a general linear model (GLM) implementation of the block-based paradigm. The estimated motion parameters from the re-alignment process were used as a regressor. Framewise displacement was calculated from the re-alignment parameters^43,44^. For this, projection to the surface of a sphere of 50 mm radius was used to convert rotational displacements from degrees into mm after motion parameter de-meaning and de-trending^44^. Activation maps were re-calculated after removing subjects that had mean FDs ≥ 0.20 mm and compared to the full data-set to test for the impact of motion on the results*.* Using the standard haemodynamic response (HRF) function, t-contrast images for all three tasks were created with an FWE-corrected *p*-value threshold of *p* = 0.05 yielding a t-contrast image for each condition.

### Conjunction analysis

The resultant contrast images (one for each condition) were then used to perform a second level conjunction (one-way ANOVA random effects) analysis across all contrast images of the individual tasks. Briefly, the steps resemble those of a general second-order analysis: All first-order contrast images (one for each subject and each condition) were added into a second-order analysis (one-way ANOVA). The images were grouped by condition. After estimation of the model, contrasts for each condition were defined as usual. To compute the joint-conjunction, multiple contrasts at a time were selected. Statistical maps were calculated with correction for multiple comparisons (FWE), a *p*-value of 0.05 and cluster extent of 20 voxels.

The tasks were also studied individually adjunct to the conjunction analysis to assess the effect of each task. These individual maps were processed in MRICron (ch2.better.niftii) and superimposed on each other.

### ROI time course analyses

A third analysis concerned comparing and contrasting activity in ROI that were functionally defined from the activation patterns seen in the conjunction analyses. These ROI were chosen because those regions have shown significant activations during resting- and task-absent periods^4,5,32,53^. Similarly, they have shown ROI-activations during active processing and task-presence^15,19,28,29,54^ including tasks and processes covered by the current three tasks.

For this time-course analysis, the ROI were created using MarsBaR v0.44^55^ from the thresholded SPM clusters. Mean voxel values over the ROI were then extracted for each subject from the motion-corrected intensity images in MNI-space. These images represent the actual BOLD signal change at each voxel, rather than the modelled, HRF-corrected response. The images were then averaged over the cohort into a single time-course curve. This resultant time-course curve was rescaled as a percentage of total BOLD signal change, see Fig. 3.
